# Multiple myeloma as a challenging multidimensional random process: a data-driven web-based application for treatment selection

**DOI:** 10.1038/s41408-025-01238-4

**Published:** 2025-02-27

**Authors:** Alexander S. Luchinin, Tigran G. Gevorkyan, Anastasia A. Semenova

**Affiliations:** The Blokhin National Medical Research Center of Oncology of the Russian Ministry of Health, Moscow, Russia

**Keywords:** Myeloma, Lymphoma

Multiple myeloma (MM) is a blood cancer disease characterized by periods of remission and progression influenced by numerous factors [1]. Current approaches of MM therapy are based on balancing the expected efficacy and toxicity of treatment to prolong duration and improve patients’ quality of life. However, methods for determining an optimal treatment strategy are imperfect [2]. Not all clinical recommendations are feasible in different healthcare settings. In this scenario, physicians tend to continue using their own experience and empirical approaches. Any recommendations require constant updating, though the pace of updating cannot keep up with changing therapeutic landscapes in MM [3].

Advancements in therapy over the past 30 years have greatly improved our ability to treat MM [4]. Despite the dozen options available, it can be challenging to directly compare all these treatment regimens due to the cost and time needed for additional clinical trials [2, 5].

The unpredictable nature of the disease’s course allows us to view myeloma as a multidimensional random process. In such a model, biological, immunological, cytogenetic, and other characteristics of the tumor and the patient’s body act as measurable parameters [1, 6]. The response to treatment is also probabilistic and influenced by many factors, making it challenging to predict effectiveness and to choose the optimal treatment. The process of treating a patient is extremely indeterministic, meaning a variety of scenarios for the disease course and response to treatment are possible [7, 8]. Usually, we react to the occurrence of a particular scenario without predicting it in advance. However, prediction is possible with varying degrees of probability, depending on our actions at the previous stage. For example, depending on the treatment we prescribe, the list of scenarios for the disease’s further development for a given patient. By increasing the likelihood of favorable scenarios, the overall effectiveness of therapy can be improved.

The problem of choosing optimal therapy for a patient with MM in current clinical practice is challenging, particularly in the relapsed setting because of the number of options available and the large number of variables that affect the choice of therapy. We can maximize the likelihood of favorable outcome by choosing the best available therapy. An urgent goal therefore is to develop a system that can be used in clinical practice, to identify the most appropriate options that should be considered for each patient. Such a system should examine the variety of existing therapeutic options utilizing available data, updated regularly, and easy to use for clinicians. It should list the most appropriate treatment options along with the published response and outcome data for each of them. We present a proposed web-based tool for selecting the most effective MM therapy that fulfils the goals listed above. This tool was developed by a team of experts from The Blokhin National Medical Research Center of Oncology of the Russian Ministry of Health, combining advanced computational methods with clinical expertise to ensure its relevance and reliability.

The web app is available at https://oncotriage.com/. It allows for the search and indirect comparison of treatment options in MM, and is presented here as an example, and not meant to be recommendations for therapy or medical advice. Users can input patient-specific information through a user-friendly interface. Following the tools output, the user gets a list of recommended treatment options ranked according to their predicted efficacy. The proposed approach considers all available data, including clinical studies and real-world evidence, in modeling the integral indicator of treatment response.

The information on treatment options and their efficiency (very good partial response rate or better (VGPR + ), progressive-free survival and minimal residual disease) used in the web app database are obtained manually from scientific medical publications indexed in the US National Library of Medicine’s MEDLINE database since 2003. Data from proceedings from the American Society of Clinical Oncology, European Hematology Association, and American Society of Hematology conferences are also incorporated. At present, the web app considers data from a total of 67,257 MM patients, including 167 therapeutic options from 384 different studies. All therapeutic approaches were marked according to indications for use: the line of therapy (1st, 2nd, 3rd or higher), the MM cytogenetic risk (high or standard), t(11;14) (to consider indications for BCL2 inhibitors), stem cell transplantation eligibility, frailty status (fit/unfit) and significant renal failure (creatinine clearance <40 mL/min/1.73 m2). Also, all studies marked by type (randomized clinical trials (RCT), single-arm clinical trials (CT), real-world evidence (RWE)) and other settings. Whenever new articles are published, the app’s database is manually updated in real-time.

The web app uses Monte Carlo simulation to calculate estimates of treatment efficacy. The method models the studied process mathematically using random variable generators. This method calculates a priori probabilities of VGPR+ with confidence intervals (CI) using the collected publications database. It sequentially models VGPR+ probability on random samples obtained by resampling from originally enrolled studies. To further support interpretation and analysis, the platform incorporates a retrieval-augmented generation (RAG)-enabled large language model (RAG-LLM). This addition allows for dynamic referencing of external knowledge to interpret simulation outcomes and analyze abstracted data from scientific literature, including toxicity profiles. This hybrid approach maximizes the reliability and clinical utility of treatment recommendations for patients with multiple myeloma (MM).

To illustrate the Monte Carlo simulation method used by the web app for producing efficacy estimates and to generate a rough ranking, here we present results for two representative treatment options across different lines of therapy: VRd (bortezomib/lenalidomide/dexamethasone) as 1^st^ line therapy and cilta-cel (ciltacabtagene autoleucel) as 3^rd^ or higher line therapy.

Based on the Monte Carlo simulation using 18 reference clinical studies (RCT – 10, CT – 1, RWE – 7) and including 4,929 total patients, the estimated VGPR+ probability for VRd as first-line therapy is 0.69 (95%CI: 0.677–0.702), see Fig. 1. For cilta-cel in third-line or higher use, the VGPR+ probability was estimated to be 0.83 (95%CI: 0.804–0.871) based on 5 studies (RCT - 3, CT - 1, RWE - 1) with 445 patients in total, see Fig. 2.

**Fig. 1:**
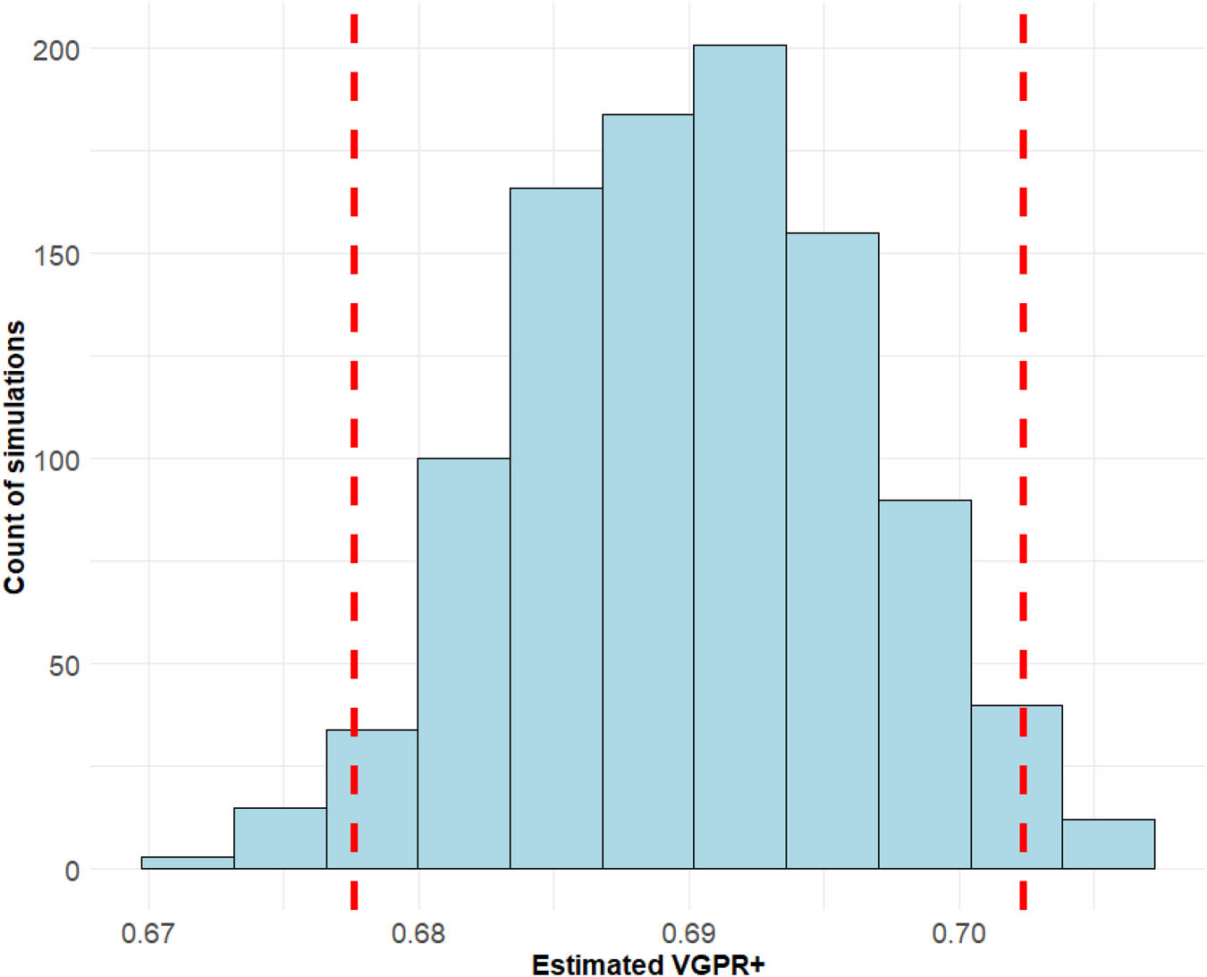
Monte Carlo simulation of VGPR+ in patients with MM using VRd as 1^st^ line of therapy.

**Fig. 2:**
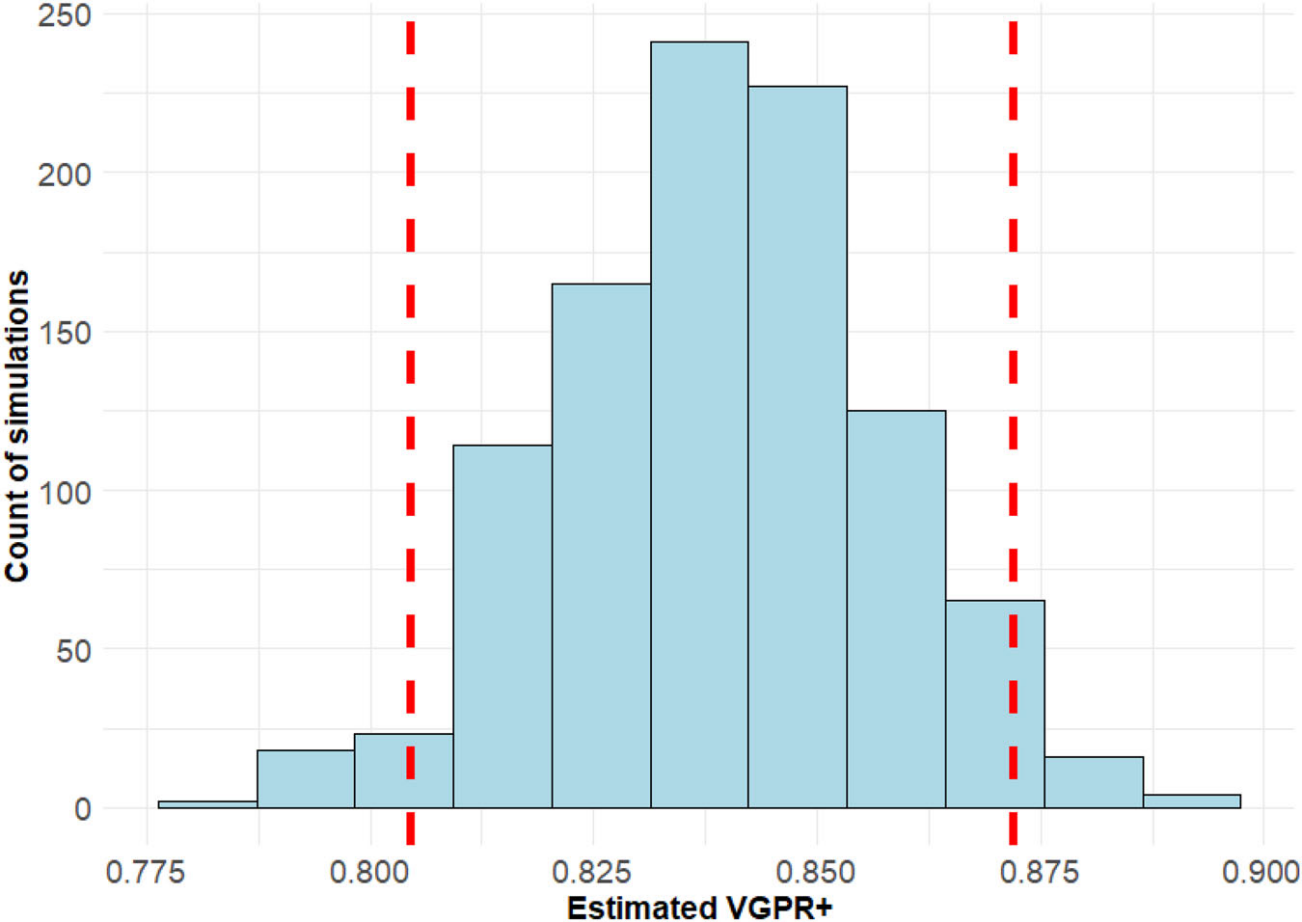
Monte Carlo simulation of VGPR+ in patients with MM using Cilta-cel as 3^rd^ or higher line of therapy.

These results demonstrate the simulation model’s ability to incorporate published evidence on diverse regimens and reliably estimate treatment-specific VGPR+ probabilities while accounting for heterogeneity between studies. Because uncertainty intervals are inversely proportional to the size of the patient sample, clinical trials with small numbers of patients may provide less reliable estimates of the probability of response to treatment.

Today hematologists are concerned with the development of risk-adapted, response-adapted and personalized treatment approaches for MM [9, 10]. One of the major challenges in choosing the most effective treatment option for MM is the large number of patient- and disease-related variables that must be considered, as well as the extremely wide variety of therapeutic options. Another problem is the lack of strict consensus regarding the optimal option for a particular clinical profile of a patient. The number of disease scenarios for a single patient, in the context of various treatment methods, can range from tens to hundreds. To date, there are no uniform rules or algorithms that allow for choosing the optimal scenario among them.

The web app we propose is a possible solution for choosing the optimal treatment regimen in MM based on the most important variables that need to be taken into consideration when making that determination. It also incorporates generative AI with retrieval-augmented generation (RAG) capabilities to enhance the interpretation of results and analyze external data, such as toxicity profiles and regional treatment availability. This integration ensures that the recommendations are grounded in both real-world evidence and up-to-date literature.

We also think it could be combined with ongoing efforts that list available treatments in each country so that the output can be tailored based on which treatments are feasible. Finally, it may serve as the backbone bone for future where the ranking could get real time adjustment based on input from myeloma experts.

Our basic evaluation shows that the web app’s output align with current clinical guidelines for MM therapy, while also incorporating new treatment options based on real-world data and the latest clinical trials. However, for final conclusions, and for clinical use we need comparative studies.

## Data Availability

This is an editorial. There is no new data generated for this manuscript, and data sharing is not applicable.
