# Correction: Multiple myeloma as a challenging multidimensional random process: a data-driven web-based application for treatment selection

**DOI:** 10.1038/s41408-025-01249-1

**Published:** 2025-03-13

**Authors:** Alexander S. Luchinin, Tigran G. Gevorkyan, Anastasia A. Semenova

**Affiliations:** The Blokhin National Medical Research Center of Oncology of the Russian Ministry of Health, Moscow, Russia

**Keywords:** Myeloma, Lymphoma

Correction to: *Blood Cancer Journal* 10.1038/s41408-025-01238-4, published online 27 February 2025

In this article the funding section has been updated from “Dr. Semenova reports no financial conflicts related to the submitted work. Dr. Gevorkyan reports no financial conflicts related to the submitted work. Dr. Luchinin reports no financial conflicts related to the submitted work.” to “This research has been financially supported by The Analytical Centre for the Government of the Russian Federation (Agreement No. 70-2024-000121 dd 29.03.2024. IGK 000000D730324P540002).

Dr. Semenova reports no financial conflicts related to the submitted work. Dr. Gevorkyan reports no financial conflicts related to the submitted work. Dr. Luchinin reports no financial conflicts related to the submitted work.”

The original article has been corrected.

